# Improvement in symptoms and signs in the forefoot of patients with rheumatoid arthritis treated with anti-TNF therapy

**DOI:** 10.1186/1757-1146-3-10

**Published:** 2010-06-17

**Authors:** Catherine J Bowen, Christopher J Edwards, Lindsey Hooper, Keith Dewbury, Madeleine Sampson, Sally Sawyer, Jane Burridge, Nigel K Arden

**Affiliations:** 1 School of Health Sciences, University of Southampton, Southampton, UK; 2 Research Development and Support Unit, University of Southampton, Southampton, UK; 3 Ultrasound Department, Department of Radiology, Southampton University Hospitals NHS Trust, Southampton General Hospital, Southampton, UK; 4 Department of Rheumatology, Southampton University Hospitals NHS Trust, Southampton General Hospital, Southampton, UK; 5 Wellcome Trust Clinical Research Facility, Southampton University Hospitals Trust, Southampton General Hospital, Southampton, UK; 6 MRC Epidemiology Resource Centre, University of Southampton, Southampton, UK; 7 NIHR Musculoskeletal Biomedical Research Unit, University of Oxford, Oxford, UK

## Abstract

**Background:**

Inhibition of tumour necrosis factor **(**TNF) is an effective way of reducing synovitis and preventing joint damage in rheumatoid arthritis (RA), yet very little is known about its specific effect on foot pain and disability. The aim of this study was to evaluate whether anti-TNF therapy alters the presence of forefoot pathology and/or reduces foot pain and disability.

**Methods:**

Consecutive RA patients starting anti-TNF therapy (infliximab, etanercept, adalimumab) were assessed for presence of synovial hypertrophy and synovitis in the 2^nd ^and 5^th ^metatarso-phalangeal (MTP) joints and plantar forefoot bursal hypertrophy before and 12 weeks after therapy. Tender MTP joints and swollen bursae were established clinically by an experienced podiatrist and ultrasound (US) images were acquired and interpreted by a radiologist. Assessment of patient reported disease impact on the foot was performed using the Manchester Foot Pain and Disability Index (MFPDI).

**Results:**

31 patients (24 female, 7 male) with RA (12 seronegative, 19 seropositive) completed the study: mean age 59.6 (SD 10.1) years, disease duration 11.1 (SD 10.5) years, and previous number of Disease Modifying Anti Rheumatic Drugs 3.0 (1.6). Significant differences after therapy were found for Erythrocyte Sedimentation Rate (t = 4.014, p < 0.001), C-reactive protein (t = 3.889, p = 0.001), 28 joint Disease Activity Score (t = 3.712, p = 0.0001), Visual Analog Scale (t = 2.735, p = 0.011) and Manchester Foot Pain and Disability Index (t = 3.712, p = 0.001).

Presence of MTP joint synovial hypertrophy on US was noted in 67.5% of joints at baseline and 54.8% of joints at twelve weeks. Presence of plantar forefoot bursal hypertrophy on US was noted in 83.3% of feet at baseline and 75% at twelve weeks. Although there was a trend for reduction in observed presence of person specific forefoot pathology, when the frequencies were analysed (McNemar) this was not significant.

**Conclusions:**

Significant improvements were seen in patient reported foot pain and disability 12 weeks after commencing TNF inhibition in RA, but this may not be enough time to detect changes in forefoot pathology.

## Background

Tumour Necrosis Factor (TNF) inhibition is known to be an effective way of reducing synovial hypertrophy and preventing erosions in patients who have rheumatoid arthritis (RA) [1,2]. Unfortunately studies to date have tended to focus on the hand joints and little is known of the effect of TNF inhibition on the foot.

Prevalence rates for pain and swelling in the feet in RA are high [3] and this correlates with patient reported outcome measures of impairment, activity limitation and participation restriction [4]. In fact, most patients with RA continue to report frequent and disabling foot pain despite pharmacological management, including TNF inhibition [5-8].

We have previously reported higher prevalence rates of forefoot pathology detectable by ultrasound (US) than by clinical examination [9]. Clinical assessment techniques are relatively insensitive in assessment of RA disease within the foot, and clinically under reported manifestations of RA within the foot appear to be a common finding in imaging studies [10-12]. Although investigators have reported the demographic of TNF inhibition, none have formally assessed the effects on foot symptoms and pathology.

US is beneficial in monitoring the progression of active inflammation in rheumatoid arthritis [13] and in the monitoring of biologic therapy [14-16]. The aim of this study was to evaluate by means of US, the effects of anti-TNF therapy in RA patients with specific focus on the forefoot using the hypothesis that forefoot symptoms, like those of hand joint symptoms, would improve with this intervention.

## Methods

A prospective study design was utilized, in which the forefeet of a consecutive cohort of patients with RA diagnosed according to the ACR criteria (modified 1987) [17] starting anti-TNF therapy (infliximab, etanercept or adalimumab) was examined clinically and by US at baseline and twelve weeks following therapy.

### Participant selection

Participants were recruited from patients with RA attending the Rheumatology

Department, Southampton University Hospitals NHS Trust who were starting anti-TNF therapy. In compliance with the declaration of Helsinki, Local Research Ethics Committee approval and informed consent was secured prior to data collection.

We enrolled consecutive patients with RA who were about to start anti-TNF therapy and were considered appropriate for this study. Our recruitment criteria excluded any patients from the study if they had a history of previous forefoot surgery, received a corticosteroid injection to the forefoot within the 3 months prior to this study, had an additional musculoskeletal disease (e.g. primary osteoarthritis, gout, Paget's disease, systemic lupus erythematosus), or had a serious medical (other than RA) or psychological disorder that would prevent completion of the study protocol.

### Data collection

Data collection took place between February 2005 and June 2007. The longer time allowed for sufficient numbers to be recruited as only a limited number of patients were starting anti-TNF therapy during that period. All clinical assessments took place within the Wellcome Trust Clinical Research Facility, Southampton General Hospital. All US scans were undertaken by a Radiologist (KD or MS) and took place in the Department of Radiology, Southampton General Hospital. On each visit, the same treatment bays and US facilities were utilised. An experienced podiatrist assessed both forefeet of all participants (CJB) and the presence of any swelling and/or tenderness was recorded. To reduce recall bias, all investigators (CJE, SB, CJB, KD and MS) were blinded to each other's results and all were blinded to their own baseline assessment of the individual patient being assessed.

### Assessment of demographic and clinical characteristics

Prior to data collection, the diagnosis of RA was confirmed by the supervising consultant (CJE). Following acceptance into the study all participants were assessed by a trained specialist rheumatology nurse (SB) and Disease Activity Scores (DAS-28) were calculated [18].

General demographic and clinical data of age, gender, disease duration, presence of rheumatoid factor, current medication, and current and previous use of Disease Modifying Anti-Rheumatic Drugs (DMARDs) were obtained from the Rheumatology Department database and clinical notes.

Clinical characteristics included visual analog scale (VAS 100 mm) assessment for the patient's global impression of health, and assessment of disease activity by the number of painful, tender and swollen joints calculated as Disease Activity Scores (DAS-28).

### Foot assessments

Foot symptoms were determined by the use of a validated patient administered index, the Manchester Foot Pain and Disability Index (MFPDI) [19]. The MFPDI asked participants to rate a series of 19 questions and took approximately five minutes for participants to complete. The forefeet of all participants were assessed by an experienced podiatrist (CJB) for MTP joint synovitis and plantar forefoot bursitis. All foot assessments were conducted at the same time as the US scans.

### Other clinical data

Laboratory assessments included blood tests for C-reactive protein (CRP) and Erythrocyte Sedimentation Rate (ESR) on the same day as the foot assessments and US scans.

### Imaging data

**Figure 1:**
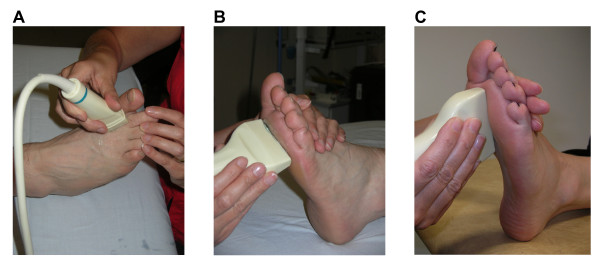
**Photographs demonstrating the dorsal longitudinal approach to assess the MTP joints (a), and the plantar transverse (b) and longitudinal (c) positions of the US transducer to assess the plantar forefoot area**.

US examinations were performed using a Philips HDI 5000 System (Royal Philips Electronics, Netherlands) in B-Mode using a 5-10 MHz linear probe. Images were recorded in two perpendicular planes, longitudinal and transverse and performed moving from proximal to distal as suggested by the EULAR (European League against Rheumatism) working group for musculoskeletal US in rheumatology guidelines [20]. A dorsal approach to detect MTP joint synovial hypertrophy, synovitis and erosion with the patient in a supine position was also adopted as recommend by the EULAR guidelines, [21] (see Figure 1a).

Observations of MTP joint synovial hypertrophy, synovitis and erosions by US (grey scale and power Doppler where possible) were conducted within just two joints in each foot (second and fifth MTP joints) from both a plantar and dorsal approach. Synovial hypertrophy, synovitis and erosions were registered as being present or absent. We have previously reported on the selection of the second and fifth MTP joints as being representative of the forefoot joints [22] and this has also been corroborated by others more recently [23]. On US synovial hypertrophy appears as hypoechoic intra-articular tissue [24] and if this is inflammatory active synovitis, it shows as a positive power Doppler US signal which gives a colour spectral map superimposed onto the grey scale image [25]. Any synovial hypertrophy, synovitis and erosion within the second and fifth MTP joints identified by US were recorded on a data sheet.

At the time of this study there was no standard definition for imaging of clinically apparent plantar forefoot bursal swelling by US. We therefore decided to use a plantar approach as we were interested in determining the prevalence of bursal hypertrophy within the plantar forefoot region. Two types of bursal hypertrophy may occur within the plantar forefoot region, intermetatarsal or sub-metatarsal. Intermetatarsal bursal hypertrophy appears on US as a well defined fluid collection with hypoechoic or anechoic zones usually bulging more than 1 mm under the metatarsal head level [10,26]. Sub-metatarsal bursal hypertrophy is attributed to adventitial bursitis and defined on US as anechoic or heterogenous collections of fluid within the sub-metatarsal fat pad [26] .

For each plantar scan the transducer was placed transversely and moved laterally from the first MTP joint with its centre at the level of the metatarsal heads (see Figure 1b). The process was repeated longitudinally (see Figure 1c). The presence of bursal hypertrophy across the plantar forefoot region identified by US was recorded on a data sheet.

### Statistical analyses

Data evaluation and statistical analysis were performed using SPSS version 17.0 software (SPSS, Chicago IL). The data was initially examined using histograms and scatter plots to identify 'outliers' that may have occurred due to data entry bias or normal biological outliers. The prevalence of MTP joint synovial hypertrophy, synovitis, erosions and plantar bursal hypertrophy per foot and per anatomical site within the forefoot is described via the mean, standard deviations and frequencies. Paired t-tests for parametric continuous data were performed to determine whether there were differences in patient reported foot pain and disability (MFPDI) between baseline and twelve weeks as well as the clinical variables, ESR, CRP, global well- being (VAS) and DAS-28. To determine change in the presence of forefoot pathology, the presence of MTP joints with synovial hypertrophy, MTP joints with synovitis, and total numbers of plantar forefoot bursal hypertrophy per foot were tested for differences using a McNemar test for categorical data of related groups.

## Results

### Participant demographics and clinical characteristics

**Figure 2:**
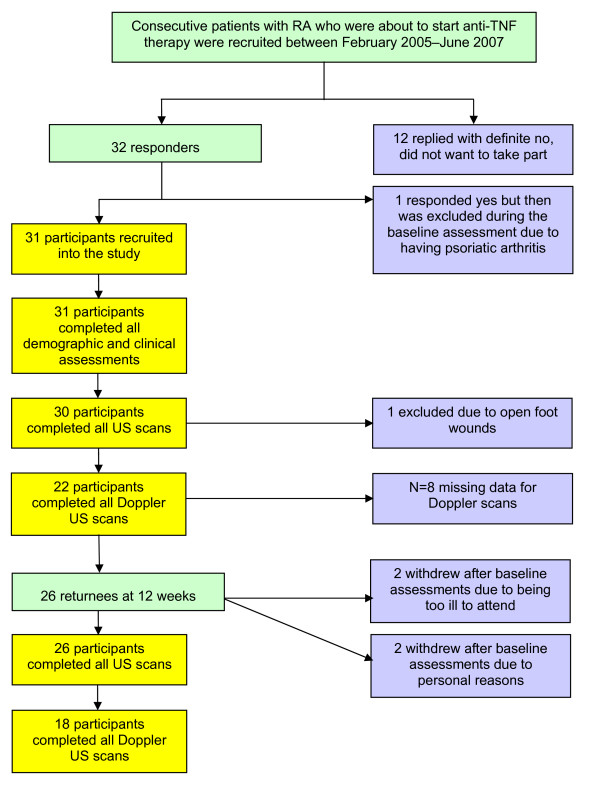
**Participant recruitment flow chart of returnees and non returnees at baseline and twelve weeks**.

From thirty two patients with RA, starting anti-TNF therapy recruited, one patient was excluded at the baseline visit due to having the wrong diagnosis and one was excluded from the US foot scans due to having open wounds on the plantar forefoot area. Four participants did not return for the ultrasound scans at the twelve week visit (see Figure 2). We did not exclude anyone at the initial participant recruitment session; however a small number of individuals did decline to take part in the study. Thirty one patients therefore started the study, 24 female and 7 male patients, 12 rheumatoid-factor negative and 19 rheumatoid-factor positive. The majority of participants had established RA with a mean age of 59.58 (SD 10.14; range 37-76) years, and duration of RA 11.1 (SD 10.52; range 1-39) years. In line with NICE guidance [27], all had previously been taking DMARDs, (mean 3.0, SD 1.6, per person). A comparison of demographic and clinical characteristics at baseline and twelve weeks can be seen in Table 1.

**Table 1:** Demographic and clinical characteristics of the study participants at baseline and twelve weeks by mean (standard deviation)

Variable	Baseline	12 weeks	Statistical Values
MFPDI (×/38)	23.17 (9.23)	17.13 (9.73)	t = 3.712, p = 0.001
VAS* (100 mm)	60.29 (21.12)	45.31 (23.25)	t = 2.735, p = 0.011
ESR (mm/hour)	37.86 (24.48)	26.5 (16.4)	t = 4.014, p < 0.001
CRP (mg/litre)	31.92 (27.15)	13.98 (15.38)	t = 3.889, p = 0.001
DAS-28	5.76 (0.93)	4.54 (1.5)	t = 3.712, p = 0.001

*Overall well being

There was a statistically significant reduction in patient reported foot pain and disability after twelve weeks of anti-TNF therapy (t = 3.712, p = 0.001). Similarly all other clinical disease measures were significantly reduced: ESR (t = 4.014, p < 0.001), CRP (t = 3.889, p = 0.001), DAS-28 (t = 3.712, p = 0.001) and global wellbeing VAS (t = 2.7351, p = 0.011).

### Prevalence of forefoot pathology in patients with RA detected by US

**Figure 3:**
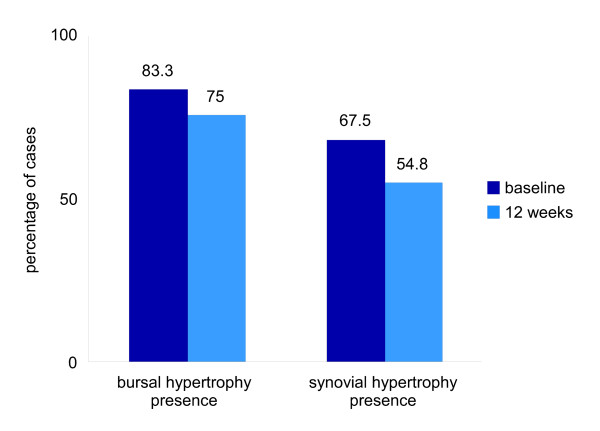
**A simple bar chart representing the percentages of cases of US detectable forefoot bursal hypertrophy and MTP joint synovial hypertrophy at baseline and following twelve weeks of anti-TNF therapy**.

At both baseline and twelve weeks a higher prevalence of forefoot pathology per individual was detected by US than by clinical examination (see Table 2). There was an observed trend for reduction in presence of person specific US detectable plantar forefoot bursal hypertrophy and MTP joint synovial hypertrophy between baseline and twelve weeks (see Figure 3).

**Table 2:** Comparison of the prevalence of MTP joint and foot specific US detectable and clinically detectable pathology of the study participants at baseline and twelve weeks by frequency (N)

Variable		Baseline	12 weeks	Statistical Values
MTP joint synovial hypertrophy (US)	R2	58.1% (18)	45.2% (14)	p = 1.000
	R5	74.2% (23)	51.6% (16)	p = 0.344
	L2	61.3% (19)	35.5% (11)	p = 0.267
	L5	67.7% (21)	54.8% (17)	p = 0.754
MTP joint synovitis (Doppler US)	R2	6.5% (2)	6.5% (2)	p = 1.000
	R5	9.7% (3)	3.2% (1)	p = 0.625
	L2	3.2% (1)	0% (0)	p = 1.000
	L5	9.7% (3)	6.5% (2)	p = 1.000
MTP joint erosion (US)	R2	25.8% (8)	25.8% (8)	p = 0.250
	R5	74.2% (23)	51.6% (16)	p = 0.727
	L2	9.7% (3)	12.9% (4)	p = 1.000
	L5	61.3% (19)	58.1% (18)	p = 1.000
Plantar forefoot bursal hypertrophy (US)	R	83.9% (26)	67.7% (21)	p = 1.000
	L	80.6% (25)	69.2% (18)	p = 0.508
Clinically detectable plantar forefoot bursae	R	41.9% (13)	32.3% (10)	p = 0.012
	L	35.5% (11)	19.4% (6)	p = 0.453
Clinically detectable MTP joint synovitis	R	45.2% (14)	25.8% (8)	p = 0.375
	L	25.8% (8)	16.1% (5)	p = 0.070

**Key**: R = Right; L = Left; 2 = second MTP joint; 5 = fifth MTP joint

Observed presence of MTP joint synovial hypertrophy by US from 60 feet (120 joints) indicates presence in 67.5% (81/120 joints) at baseline and 54.8% (57/104 joints) at twelve weeks. At twelve weeks 25.9% (27/104 joints) had changed from MTP joint synovial hypertrophy being present to absent, and 13.5% (14/104 joints) changed from MTP joint synovial hypertrophy being absent to present.

For presence of synovitis detectable by US Doppler, from 44 feet (N = 16 data missing) 10.2% (9/88 joints) was noted at baseline and 6.9% (5/72 joints from 36 feet was noted at twelve weeks. Of these, 11.1% (8/72 joints) had changed from MTP joint synovitis being present to absent and small number, 5.6% (4/72 joints) had changed from MTP joint synovitis being absent to present at twelve weeks.

Frequency of observed presence of US detectable plantar forefoot bursal hypertrophy was higher than that noted in MTP joint synovial hypertrophy but changes followed a similar pattern. US detectable forefoot bursal hypertrophy was noted in 83.3% (50/60) of feet at baseline and 75% (39/52) of feet at twelve weeks. At twelve weeks 19.2% (10/52) of feet had changed from US detectable forefoot bursal hypertrophy being present to absent and 9.6% (5/52) changed from US detectable forefoot bursal hypertrophy being absent to present.

When the frequencies for joint specific and foot specific presence of US and clinically detectable MTP joints with synovial hypertrophy, MTP joints with synovitis, and plantar forefoot bursal hypertrophy per individual were analysed no significant differences were found between the baseline data and twelve weeks (Table 2).

## Discussion

To our knowledge, our results are the first to show that patient reported foot pain and disability reduces significantly following TNF inhibition. Furthermore, using US, our results indicate that there is a trend towards reduction in US detectable MTP joint synovial hypertrophy, synovitis and plantar forefoot bursal hypertrophy following twelve weeks of TNF inhibition.

The trend towards improvement was also noted in the clinical and laboratory assessments of RA disease status (ESR, CRP and DAS-28). These reductions were statistically significant demonstrating that TNF inhibition was effective in controlling the disease process in the RA participants within this study, consistent with previous reports [1,2].

Pragmatically, whilst patient reported foot symptoms did significantly reduce, it may be hypothesized that treatment switches off the disease process of RA, but twelve weeks was not enough time for sonographic evidence of MTP joint synovitis, synovial hypertrophy and plantar bursal hypertrophy to regress within the forefeet. In previous work, within the MCP joints of the hands a reduction in sonographically detectable synovial hypertrophy was noted at eighteen weeks following infliximab therapy, and this was reported as a significant reduction at 110 weeks [2]. In explaining US detection of synovitis within joints, Brown et al [28] report that synovium may become chronically thickened and less reversible in established disease. This was typically demonstrated within our sample in which the majority of participants had established RA with a mean duration of 11.1 years. Using Doppler US 11.1% of MTP joints examined that showed a positive US Doppler response at baseline had none after twelve weeks, yet synovial hypertrophy remained evident on US in 54.8% of MTP joints at twelve weeks. This is consistent with a recent study where 42 joints (bilateral glenohumeral, elbows, wrists, MCPs, proximal interphalangeal, knees, tibiotalar, midtarsal and MTPs) were assessed with the most common joints with US detectable synovitis being the wrist, hands and feet and power Doppler US signal occurring less frequently in the MTP joints [29].

Foot symptoms were not inclusion criteria within our study, yet the high prevalence detectable by US was surprising. There are suggestions that clinically evident disease improves more quickly following effective treatment than disease assessed by modern imaging techniques [11]. In a previous study of patients treated with anti-TNF therapy, 90% demonstrated clinical remission at week 14, but none had absence of imaging synovitis [29]. We can infer this from our findings too, whereby a much higher prevalence of US detectable plantar MTP joint synovial hypertrophy and plantar forefoot bursal hypertrophy was evident than that detectable by clinical examination at both baseline and twelve weeks.

Of further note, in a minority of participants, clinical disease activity and well being scores improved but the prevalence of US detectable MTP joint synovial hypertrophy and plantar forefoot bursal hypertrophy increased. This anomalous finding could be attributable to imperfect reproducibility of the US measurements, a reported phenomenon in the use of US [30], although as technology has improved reliability in US techniques have improved [31]. Alternatively, although the MFPDI improves with treatment, the questionnaire is developed for foot complaints in general and does not focus on the forefoot [19]. Therefore it is possible that the difference between self-reported symptoms in US detectable signs in the forefoot may also be attributable to a faster recovery of other foot joints than the MTP joints. Increased mechanical stresses, possibly as mobility improved for those patients, rather than an increase in episodes of synovial hypertrophy and bursal hypertrophy due to the disease process of RA may also be the cause of prolonged US detectable synovial hypertrophy within the forefeet.

Mechanical and gait data would have been useful to explore this concept further. van der Leeden, Steultjens et al [32] demonstrated correlations of radiographic MTP joint deformity with peak pressure and pressure time integrals for the first and fourth MTP joints and correlations of high forefoot pressures with pain in RA participants. In a later systematic review of foot related measures, van der Leeden, Steultjens et al [33] recommend considering both self report and performance based instruments when investigating foot problems associated with RA. We chose to focus on clinical assessment of foot pathology, foot disability and foot pain and no account was taken of the mechanical forces of foot function during gait as this was not feasible within the confines of our study. Others, having investigated the association between foot disabilities, mechanics of function and foot mechanics, recommend that future prediction models may be enhanced by combining imaging based identification of foot pathology with mechanical data [4].

No techniques have yet been developed to detect differences between mechanically related inflamed hypertrophied synovium and active inflammatory synovium related to disease activity in RA within the foot. Brown, Conaghan et al [28] highlight that grey scale US primarily detects hypertrophy of the synovium but does not differentiate between inflammatory and non inflammatory synovitis. For future studies the further use of power Doppler US, Gadolinium enhanced MRI and/or biopsy for histological analysis of forefoot pathology would be needed to achieve this as well as the development of a technique that includes the effect of foot mechanics.

This study has several strengths and a number of potential limitations. Strengths include that it was a pragmatic clinical study representative of secondary care in the UK. In addition, a sample of patients with RA at the same stage of treatment was investigated. Furthermore, patient reported clinical outcomes including disease activity and foot specific measures were used.

Potential limitations include the fact the presence of US detectable MTP joint synovitis, synovial hypertrophy and bursal hypertrophy within the forefoot was not validated by any other 'gold standard' imaging technique, such as Magnetic Resonance Imaging (MRI) or by histological analysis through biopsy. Soft tissue swelling at the level of the MTP joints in the plantar forefoot area can be related to other pathology such as tenosynovitis or rheumatoid nodules that could be better differentiated using MRI [34,35]. Due to its restricted availability MRI was not feasible for our study. Others have attempted to validate imaging findings using fresh cadavers [36] however this technique was also not a feasible option during our clinical study.

Limitations relating to the sample and time frame also have to be highlighted. The sample was relatively small; therefore generalizibility to the whole population of patients with RA needs to be confirmed. The time frame of twelve weeks gave only two points of assessment over a relatively short period. Although providing useful information, more time points over a longer time period would have allowed variations in individual foot states to be monitored more effectively.

## Conclusions

We have demonstrated that patient reported foot pain and disability does reduce significantly following twelve weeks of TNF inhibition. We have also provided some evidence that suggests forefoot pathology in RA may improve following a short period of TNF inhibition; however further evidence over a longer time period is required to confirm that this is sustained. The findings further indicate that the use of US imaging of the foot would be more beneficial than clinical examination alone in the refinement of diagnosis and the implementation of effective care pathways for patients who have foot symptoms and are starting TNF inhibition.

## List of abbreviations

TNF: Tumour necrosis factor; RA: Rheumatoid arthritis; MTP: Metatarso-phalangeal; US: Ultrasound; MFPDI: Manchester foot pain and disability index; SD: Standard deviation; DMARDs: Disease modifying anti-rheumatic drugs; ESR: Erythrocyte sedimentation rate; CRP: C-reactive protein; DAS-28: Disease activity score of 28 swollen and tender joints; VAS: Visual analog scale; ACR: American College of Rheumatology; EULAR: European League Against Rheumatism; NICE: The National Institute for Health and Clinical Excellence; MRI: Magnetic resonance imaging.

## Competing interests

The authors declare that they have no competing interests.

## Authors' contributions

CB conceived of the study, carried out the ultrasound foot study of patients with RA treated with anti-TNF therapy, participated in the clinical assessment of foot status and drafted the manuscript. CE participated in the design of the study, carried out the assessment of eligibility of RA patients for anti-TNF therapy and helped to draft the manuscript. LH participated in the data analysis and helped to draft the manuscript. KD and MS participated in the design of the study and performed the US assessments. SS participated in the study design and coordination. JB participated in the design of the study and helped to draft the manuscript. NA participated in the conception and design of the study and helped to draft the manuscript. All authors read and approved the final manuscript.

## References

[B1] Hau M Kneitz C Tony HP Keberle M Jahns R Jenett M High resolution ultrasound detects a decrease in pannus vascularisation of small finger joints in patients with rheumatoid arthritis receiving treatment with soluble tumour necrosis factor alpha receptor (etanercept) Ann Rheum Dis 2002 61 55 58 10.1136/ard.61.1.55 11779760 PMC1753871

[B2] Taylor PC Steuer A Gruber J McClinton C Cosgrove DO Blomley MJ Marsters PA Wagner CL Maini RN Ultrasonographic and radiographic results from a two-year controlled trial of immediate or one-year-delayed addition of infliximab to ongoing methotrexate therapy in patients with erosive early rheumatoid arthritis Arthritis Rheum 2006 54 47 53 10.1002/art.21544 16385521

[B3] van der Leeden M Steultjens MP Ursum J Dahmen R Roorda LD Schaardenburg DV Dekker J Prevalence and course of forefoot impairments and walking disability in the first eight years of rheumatoid arthritis Arthritis Rheum 2008 59 1596 1602 10.1002/art.24188 18975350

[B4] Turner DE Helliwell PS Siegel KL Woodburn J Biomechanics of the foot in rheumatoid arthritis: identifying abnormal function and the factors associated with localised disease 'impact' Clin Biomech (Bristol, Avon) 2008 23 93 100 10.1016/j.clinbiomech.2007.08.009 17904711

[B5] Rojas-Villarraga A Bayona J Zuluaga N Mejia S Hincapie ME Anaya JM The impact of rheumatoid foot on disability in Colombian patients with rheumatoid arthritis BMC Musculoskelet Disord 2009 10 67 10.1186/1471-2474-10-67 19527518 PMC2702313

[B6] Rome K Gow PJ Dalbeth N Chapman JM Clinical audit of foot problems in patients with rheumatoid arthritis treated at Counties Manukau District Health Board, Auckland, New Zealand J Foot Ankle Res 2009 2 16 10.1186/1757-1146-2-16 19442310 PMC2685775

[B7] Otter SJ Lucas K Springett K Moore A Davies K Cheek L Young A Walker-Bone K Foot pain in rheumatoid arthritis prevalence, risk factors and management: an epidemiological study Clin Rheumatol 29 255 271 10.1007/s10067-009-1312-y 19997766

[B8] Grondal L Tengstrand B Nordmark B Wretenberg P Stark A The foot: still the most important reason for walking incapacity in rheumatoid arthritis: distribution of symptomatic joints in 1,000 RA patients Acta Orthop 2008 79 257 261 10.1080/17453670710015067 18484253

[B9] Bowen CJ Culliford D Dewbury K Sampson M Burridge J Hooper L Edwards CJ Arden NK The clinical importance of ultrasound detectable forefoot bursae in rheumatoid arthritis Rheumatology (Oxford) 2010 in press 19797311 10.1093/rheumatology/kep307

[B10] Koski JM Ultrasound detection of plantar bursitis of the forefoot in patients with early rheumatoid arthritis J Rheumatol 1998 25 229 230 9489811

[B11] Brown AK Quinn MA Karim Z Conaghan PG Peterfy CG Hensor E Wakefield RJ O'Connor PJ Emery P Presence of significant synovitis in rheumatoid arthritis patients with disease-modifying antirheumatic drug-induced clinical remission: evidence from an imaging study may explain structural progression Arthritis Rheum 2006 54 3761 3773 10.1002/art.22190 17133543

[B12] Wakefield RJ Freeston JE O'Connor P Reay N Budgen A Hensor EM Helliwell PS Emery P Woodburn J The optimal assessment of the rheumatoid arthritis hindfoot: a comparative study of clinical examination, ultrasound and high field MRI Ann Rheum Dis 2008 in press 10.1136/ard.2007.079947 18258710

[B13] McNally EG Ultrasound of the small joints of the hands and feet: current status Skeletal Radiol 2008 37 99 113 10.1007/s00256-007-0356-9 17712556 PMC2141652

[B14] Iagnocco A Perella C Naredo E Meenagh G Ceccarelli F Tripodo E Basili S Valesini G Etanercept in the treatment of rheumatoid arthritis: clinical follow-up over one year by ultrasonography Clin Rheumatol 2008 27 491 496 10.1007/s10067-007-0738-3 17952483

[B15] Saleem B Brown AK Keen H Nizam S Freeston J Karim Z Quinn M Wakefield R Hensor E Conaghan PG Emery P Disease remission state in patients treated with the combination of tumor necrosis factor blockade and methotrexate or with disease-modifying antirheumatic drugs: A clinical and imaging comparative study Arthritis Rheum 2009 60 1915 1922 10.1002/art.24596 19565512

[B16] Haavardsholm EA Ostergaard M Hammer HB Boyesen P Boonen A van der Heijde D Kvien TK Monitoring anti-TNFalpha treatment in rheumatoid arthritis: responsiveness of magnetic resonance imaging and ultrasonography of the dominant wrist joint compared with conventional measures of disease activity and structural damage Ann Rheum Dis 2009 68 1572 1579 10.1136/ard.2008.091801 19019893

[B17] Arnett FC Edworthy SM Bloch DA McShane DJ Fries JF Cooper NS Healey LA Kaplan SR Liang MH Luthra HS The American Rheumatism Association 1987 revised criteria for the classification of rheumatoid arthritis Arthritis Rheum 1988 31 315 324 10.1002/art.1780310302 3358796

[B18] Prevoo ML van't Hof MA Kuper HH van Leeuwen MA van de Putte LB van Riel PL Modified disease activity scores that include twenty-eight-joint counts. Development and validation in a prospective longitudinal study of patients with rheumatoid arthritis Arthritis Rheum 1995 38 44 48 10.1002/art.1780380107 7818570

[B19] Garrow AP Papageorgiou AC Silman AJ Thomas E Jayson MI Macfarlane GJ Development and validation of a questionnaire to assess disabling foot pain Pain 2000 85 107 113 10.1016/S0304-3959(99)00263-8 10692609

[B20] Backhaus M Burmester GR Gerber T Grassi W Machold KP Swen WA Wakefield RJ Manger B Guidelines for musculoskeletal ultrasound in rheumatology Ann Rheum Dis 2001 60 641 649 10.1136/ard.60.7.641 11406516 PMC1753749

[B21] Scheel AK Schmidt WA Hermann KG Bruyn GA D'Agostino MA Grassi W Iagnocco A Koski JM Machold KP Naredo E Interobserver reliability of rheumatologists performing musculoskeletal ultrasonography: results from a EULAR "Train the trainers" course Ann Rheum Dis 2005 64 1043 1049 10.1136/ard.2004.030387 15640263 PMC1755572

[B22] Bowen CJ Dewbury K Sampson M Sawyer S Burridge J Edwards CJ Arden NK Musculoskeletal ultrasound imaging of the plantar forefoot in patients with rheumatoid arthritis: inter-observer agreement between a podiatrist and a radiologist J Foot Ankle Res 2008 1 5 10.1186/1757-1146-1-5 18822149 PMC2553775

[B23] Backhaus M Ohrndorf S Kellner H Strunk J Backhaus TM Hartung W Sattler H Albrecht K Kaufmann J Becker K Evaluation of a novel 7-joint ultrasound score in daily rheumatologic practice: a pilot project Arthritis Rheum 2009 61 1194 1201 10.1002/art.24646 19714611

[B24] O'Connor PJ Grainger AJ Ultrasound imaging of joint disease Imaging 2002 14 188 201

[B25] Balint PV Mandl P Kane D "All that glistens is not gold"--separating artefacts from true Doppler signals in rheumatological ultrasound Ann Rheum Dis 2008 67 141 142 10.1136/ard.2007.081554 18192305

[B26] Gregg JM Schneider T Marks P MR imaging and ultrasound of metatarsalgia--the lesser metatarsals Radiol Clin North Am 2008 46 1061 1078 vi-vii 10.1016/j.rcl.2008.09.004 19038613

[B27] NICE Clinical Guidelines for Adalimumab, etanercept and infliximab for the treatment of rheumatoid arthritis National Institute for Clinical Excellence 2007 London

[B28] Brown AK Conaghan PG Karim Z Quinn MA Ikeda K Peterfy CG Hensor E Wakefield RJ O'Connor PJ Emery P An explanation for the apparent dissociation between clinical remission and continued structural deterioration in rheumatoid arthritis Arthritis Rheum 2008 58 2958 2967 10.1002/art.23945 18821687

[B29] Wakefield RJ Freeston JE Hensor EM Bryer D Quinn MA Emery P Delay in imaging versus clinical response: a rationale for prolonged treatment with anti-tumor necrosis factor medication in early rheumatoid arthritis Arthritis Rheum 2007 57 1564 1567 10.1002/art.23097 18050231

[B30] Joshua F Lassere M Bruyn GA Szkudlarek M Naredo E Schmidt WA Balint P Filippucci E Backhaus M Iagnocco A Summary findings of a systematic review of the ultrasound assessment of synovitis J Rheumatol 2007 34 839 847 17407235

[B31] Cheung P Ruyssen-Witrand A Gossec L Paternotte S Le Bourlout C Mazieres M Dougados M Reliability of clinical self-evaluation of swollen and tender joints in rheumatoid arthritis: A comparison study with ultrasonography, physician and nurse assessments Arthritis Care Res 2010 in press 10.1002/acr.20178 20235213

[B32] van der Leeden M Steultjens M Dekker JH Prins AP Dekker J Forefoot joint damage, pain and disability in rheumatoid arthritis patients with foot complaints: the role of plantar pressure and gait characteristics Rheumatology (Oxford) 2006 45 465 469 10.1093/rheumatology/kei186 16287922

[B33] van der Leeden M Steultjens MP Terwee CB Rosenbaum D Turner D Woodburn J Dekker J A systematic review of instruments measuring foot function, foot pain, and foot-related disability in patients with rheumatoid arthritis Arthritis Rheum 2008 59 1257 1269 10.1002/art.24016 18759256

[B34] Ashman CJ Klecker RJ Yu JS Forefoot pain involving the metatarsal region: differential diagnosis with MR imaging Radiographics 2001 21 1425 1440 11706214 10.1148/radiographics.21.6.g01nv071425

[B35] Studler U Mengiardi B Bode B Schottle PB Pfirrmann CW Hodler J Zanetti M Fibrosis and adventitious bursae in plantar fat pad of forefoot: MR imaging findings in asymptomatic volunteers and MR imaging-histologic comparison Radiology 2008 246 863 870 10.1148/radiol.2463070196 18195378

[B36] Theumann NH Pfirrmann CW Chung CB Mohana-Borges AV Haghighi P Trudell DJ Resnick D Intermetatarsal spaces: analysis with MR bursography, anatomic correlation, and histopathology in cadavers Radiology 2001 221 478 484 10.1148/radiol.2212010469 11687693

